# Commonalities and differences in the mutational signature and somatic driver mutation landscape across solid and hollow viscus organs

**DOI:** 10.1038/s41388-023-02802-7

**Published:** 2023-08-12

**Authors:** Aik Seng Ng, Dedrick Kok Hong Chan

**Affiliations:** 1 Radcliffe Department of Medicine, John Radcliffe Hospital, University of Oxford, Oxford, UK; 2 Department of Surgery, Yong Loo Lin School of Medicine, National University of Singapore, Singapore, Singapore; 3 Division of Colorectal Surgery, University Surgical Cluster, National University Hospital, Singapore, Singapore; 4 NUS Centre for Cancer Research, Yong Loo Lin School of Medicine, National University of Singapore, Singapore, Singapore; 5 Nuffield Department of Surgical Sciences, University of Oxford, Oxford, UK

**Keywords:** Genetic variation, Tumour heterogeneity

## Abstract

Advances in sequencing have revealed a highly variegated landscape of mutational signatures and somatic driver mutations in a range of normal tissues. Normal tissues accumulate mutations at varying rates ranging from 11 per cell per year in the liver, to 1879 per cell per year in the bladder. In addition, some normal tissues are also comprised of a large proportion of cells which possess driver mutations while appearing phenotypically normal, as in the oesophagus where a majority of cells harbour driver mutations. Individual tissue proliferation and mutation rate, unique mutagenic stimuli, and local tissue architecture contribute to this highly variegated landscape which confounds the functional characterization of driver mutations found in normal tissue. In particular, our understanding of the relationship between normal tissue somatic mutations and tumour initiation or future cancer risk remains poor. Here, we describe the mutational signatures and somatic driver mutations in solid and hollow viscus organs, highlighting unique characteristics in a tissue-specific manner, while simultaneously seeking to describe commonalities which can bring forward a basic unified theory on the role of these driver mutations in tumour initiation. We discuss novel findings which can be used to inform future research in this field.

## Introduction

Somatic mutations are caused by spontaneous genetic alterations acquired throughout life. For example, these mutations may occur as a function of aging, may be modulated by exposure to exogenous mutagens [1] such as cigarette smoke and ultraviolet radiation, or may be caused by endogenous defects in the DNA repair machinery [2] leading to DNA mismatches and replication-associated errors. In the last decade, it has been demonstrated that aetiologic causes of mutation can exhibit a distinct and characteristic mutational profile known as its mutational signature. The accumulation of mutational signatures in different tissues therefore reflects the landscape of insults leading to an accumulation of somatic mutations. Each mutational signature implies that mutations do not appear randomly in the genome, but manifest at unique genomic nucleotide settings, depending on its aetiology. Mutational signatures have most recently been updated in the ‘Catalogue Of Somatic Mutations In Cancer’ (COSMIC) database (COSMICv3.3, available https://cancer.sanger.ac.uk/signatures/) [3]. The authors sequenced 23,839 samples from a range of tissue types to uncover patterns in single-base substitutions (SBS), double-base substitutions (DBS) and indels (ID) which collectively depict the landscape of mutational signatures in tissues. Although 96 possible contexts exist, 72 SBS signatures have been described. In addition, 11 DBS and 18 ID signatures have also been described. In this review, we have updated mutational signature assignments to reflect the most recent COSMIC nomenclature to facilitate comparisons across different tissue types.

Mutational signatures give rise to somatic mutations. Somatic mutations may be synonymous or nonsynonymous. Synonymous mutations which do not result in protein sequence changes are believed to be functionally similar to their wildtype counterpart and are evolutionarily silent. In contrast, nonsynonymous mutations entail a change in the protein sequence, and are therefore subject to evolutionary selection pressures [4]. Inherent tumour heterogeneity present in most tissues reflect the natural selection which these mutations are subject to, and likely play a crucial role in disease pathogenesis [5, 6]. The accumulation of somatic mutations has also been shown to manifest in known cancer driver mutations. Given our incomplete understanding of the processes which underlie tumour initiation, the role of such driver mutations in normal tissues remains cryptic. Driver mutations in normal tissue fall into three possible categories. Firstly, they could be bystander mutations which have no inherent role in oncogenesis. Otherwise, they could be crucial initiating mutations which allow cancer to arise from such cells harbouring these mutations in a cell autonomous fashion. Lastly, mutations could bear a competitive or collaborative effect on neighbouring cells and be responsible for a field-change effect which causes the initiation of cancer in neighbouring cells.

Many excellent reviews have been written describing the mutational signatures in detail [7, 8]. In this review, we instead discuss mutational signatures insofar as they give rise to somatic mutations which portend a clonal advantage in normal tissues. We focus on the presence of somatic mutations in normal tissues, and discuss the possible roles of such somatic mutations in cancer initiation. We first provide an overview of the relationship between mutational signatures and the acquisition of somatic driver mutations. We then explore the landscape of mutational signatures and somatic driver mutations in a range of solid and hollow viscous organs, describing mutations known to harbour in such tissues, and appraise their relevance to specific cancers. We also seek to discuss and speculate on the patterns of somatic driver mutations in the range of normal tissues, and explore the roles of such mutations in cancer initiation. Finally, we consider clinically relevant potential applications which harness the characteristics of driver mutations in normal tissue, and consider how this could bring therapeutic interventions to detect cancer.

## Mutational signatures are associated with unique aetiologies

Mutational signatures can be associated with specific aetiologies. Extensive, and in-depth sequencing led to the discovery of an important group of mutational signatures which follow an age-related dynamic instead of the standard episodic accumulation of mutations and are therefore termed clock-like [9]. These comprise SBS 1, 5 and 18. These signatures could represent the neutral drift accumulation of somatic mutations in tissues. Notably, SBS 1 mutations, associated with the deamination of 5-methylcytosines, dominate in cancer types which have a high cellular turnover, such as in the stomach and colorectum, strongly suggesting that cell division and proliferation are implicated in this signature. In the stomach, the frequency of SBS 1 mutations was 23.7 mutations/Gb/year, while in the colorectum, the frequency was 23.4. This contrasts with melanoma and myeloma, in which SBS 1 frequency was only 3.2 and 3.1 respectively. SBS 2 is another signature which provides aetiologic clues. SBS 2 is characterised by C > T and C > G mutations and are found at TpCpN trinucleotides. These have been attributed to overactivity of the APOBEC cytidine deaminase which convert cytidine bases to uracil [10, 11]. Similarly, SBS 13 is also associated with APOBEC cytidine deaminases, SBS4 is associated with tobacco exposure and therefore found in liver, bronchus, and oesophageal tissue, while SBS22, associated with aristolochic acid can be found in the liver, urothelium, esophagus and duodenum [12]. The presence of mutational signatures in normal tissue strongly implies that the aetiologic processes which give rise to cancer can be traced back to a period in the tissue’s history when it was still phenotypically normal. This finding could have profound implications on our ability to detect tissues at risk for future cancer even while it remains phenotypically normal.

There however remains a large subset of mutational signatures which hitherto are not associated with any specific aetiologies. SBS 5, described above as being clock-like, is not associated with any specific form of DNA insult. Other examples of mutational signatures which are not associated with an aetiology include SBS 8, 12, 16 and 17.

## Mutational signatures may be amenable to targeted therapy

In cancer, unique mutational signatures have been shown to be amenable to targeted therapy. SBS 3 is related to biallelic inactivation of *BRCA1* or *BRCA2*, and is implicated in defective homologous recombination (HR). Breast cancers harboring this signature exhibit susceptibility and sensitivity to poly-ADP-ribose (PARP) inhibitors and may benefit from targeted PARP inhibition [13, 14]. Similarly, the efficacy of ataxia telangiectasia and Rad3-related kinase (ATR) inhibition in cancers with APOBEC-associated SBS 2 and 13 has also been demonstrated. ATR inhibition blocks the stalling of abnormal replication forks caused by APOBEC-induced mutations, therefore averting replication catastrophe [15]. While there is at present no indication to apply such forms of targeted therapy in the setting of normal tissues even if they bear a targetable mutational signature, it is encouraging to note that the accumulation of mutations need not be an immutable phenomenon. It is possible that future indications for treatment rest not on a diagnosis of cancer per se, but instead on crossing a threshold of mutational burden, even if the tissue at that point was still phenotypically normal.

## Mutational signatures give rise to somatic mutations in driver genes

Although cancers reflect significant intratumoral heterogeneity, all cancers can be evolutionarily traced to a progenitor origin where the first cell acquires a somatic mutation, conferring these cells with selective advantages over neighboring wildtype cells. Mutational signatures therefore provide insights into how these somatic driver mutations may be first acquired in phenotypically normal tissue, placing the cell on an evolutionary trajectory towards cancer [16]. Furthermore, there is evidence that the effects of early initiating events in stem cells propagate into more differentiated cells. In paired sequencing of stem and differentiated cells of the colon, mutational burdens and signatures were similar, in spite of the time lag which stem cells require to achieve clonality and manifest in differentiated cells [17]. These findings suggest that mutational processes occurring in normal stem cells can have important implications on later mutational burden in a tumour.

While all driver mutations are somatic mutations, the converse is untrue, and not all somatic mutations drive tumorigenesis, or are driver mutations. In the benign prostate, prostatic epithelial cells accumulate mutations at a rate of 16.4 mutations per clone per year, resulting in a genome-wide mutation burden of 1000 to 1500 mutations by the seventh decade of life [18]. Yet, driver mutations remain rare in spite of the high mutational burden with only one gene, *FOXA1*, being associated with clonal expansion in normal prostatic tissue. The converse is observed in the normal oesophagus, where close to 96% of normal epithelium harbour driver mutations [19]. Ultimately, this points to our inability to explain how mutational signatures translate in a tissue-specific way to the driver mutations found in them.

## The pattern of somatic driver mutations is complex across different tissues

The landscape of somatic driver mutations in normal tissues reveal a complex landscape which requires a tissue-specific approach [12]. Tissues acquire somatic mutations at differing rates. Massively parallel sequencing of normal brain frontal cortex, and colonic epithelium across a spectra of ages showed that although nuclear mutation prevalence in different tissues was similar in children, this diverged by adolescence, and markedly differed by the time of old age (childhood: colon vs brain, 1.8 ± 0.5 × 10^–7^ vs 1.1 ± 0.3 × 10^–7^, *p* > 0.05; adolescence: 5.5 ± 1.6 × 10^–7^ vs 2.2 ± 1.1 × 10^–7^, *p* < 0.05; old age: 1.1 ± 0.2 × 10^–6^ vs 6.3 ± 2.3 × 10^–7^, *p* < 0.01) [20]. This was further corroborated in a separate analysis of mutations rates found in normal tissues of the colon, small intestine, liver and skin [21]. While mutation rates differ between tissues, the rate of mutation within each tissue type remains relatively constant throughout life, suggesting that age-related mutational processes are responsible for the acquisition of a majority of somatic mutations, many of which are not related to exogenous stimuli, and therefore are unavoidable. Specific age-related mutation processes are therefore tissue specific. In a comparison among normal colon, small intestine and liver adult stem cells, stem cells were found to possess tissue-specific mutational processes [22]. In the colon and small intestine, SBS 1 formed the majority of somatic mutations, but was rare in liver stem cells. Instead, liver stem cells accumulated mutations bearing the marks of SBS 5. The dominance of signature 1 has been attributed to cells which undergo high turnover. Furthermore, in the colon and small intestine, SBS 1 mutations occur predominantly in late replicating DNA, when DNA replication occurs prior to successful DNA repair. These findings suggest that not only are there tissue specific differences in mutational processes, that mutations can occur in a biased fashion within the tissue type, and may therefore accumulate in specific regions of the genome.

It is therefore unsurprising to find a myriad of somatic driver mutations unique to each tissue type, with an incomplete overlap between tissue types. In a study which sampled normal tissue from nine organ sites from 5 individuals, a number of cancer genes which represent key cancer pathways were found to be recurrently mutated. These included *ARID1A*, *TP53*, *NOTCH1* and *FAT1*. *NOTCH1* was the most frequently mutated gene among all samples from different sites and donors. Conversely, some mutations were found exclusively in specific tissue types. Examples include *AXIN2* in the colon, *SMARCA4* in the duodenum, and *KMT2D* in the liver [12].

Other somatic driver mutations may be found both in normal tissue and cancer, or exclusively in either normal tissue or cancer. An approach which demonstrates the heterogeneity of somatic mutations in different tissues considers the overlap between cancer-associated mutations and mutations present in a pre-cancerous state. This revealed a spectrum which ranged from 100% overlap in sun-exposed skin, to low overlaps in organs such as the spleen and brain [23]. In a study which performed whole exome sequencing of matched normal tissue and cancer from a range of tissues of 392 patients, *PIK3CA* mutations were most commonly found in both cancer and normal tissue. In addition, the same variant of *PIK3CA* mutation was found in two-thirds of matched samples [24]. In contrast, *MUC6* is an example of a mutation which is present only in normal gastric tissue but uncommon in gastric cancer [12]. Intriguingly, approximately 40% of all colorectal cancers carry a *KRAS* mutation [23, 24] but it is infrequently detected in normal colonic epithelium. Conversely, prevalence of *KRAS* mutations is higher in histologically normal endometrium compared to corresponding cancer [25]. This suggests that the same mutation is likely to have variable roles depending on the tissue-specific context.

Differences in the accrual of driver mutations in tissues can be accounted for by unique tissue architectural constraints imposed on clone expansion. Blood cells are unrestricted spatially, and can therefore expand in proportion to mutational load. In contrast, colonic crypts have highly organized structures which spatially limit clonal expansion, resulting in only 1% of normal colonic epithelial cells harbouring driver mutations. In the setting of inflammatory bowel disease, disruption to this tightly ordered crypt structure allows for cells to undergo clone expansion much more readily [25]. The ability for somatic mutations in normal tissue to clonally expand is a reflection of the spatial constraints wrought by its unique tissue architecture. Li et al. quantify this by calculating the ratio of an independent index to the average mutant cell fraction (MCF) [10]. The independent index is derived by taking the ratio of the number of samples which do not share any mutation clusters divided by the total number of samples with at least one mutation cluster. Clonal expansion was defined by having a low independent index but a high MCF. In this way, tissues which were spatially constrained included the colon, rectum and duodenum while organs which were less restrained included the esophagus, and liver, broadly reflecting the anatomical constraints faced by these organs.

Taken together, these findings suggest the importance of adopting a tissue-specific approach in characterizing the acquisition of somatic mutations in normal epithelia. In the following sections, we review the literature surrounding somatic mutations relating to specific sold and hollow viscous organs.

## Tissue-specific mutational signature and somatic mutation landscape

### Breast

Genomic studies on breast tissue have revealed that most breast cancer driver genes are somatically mutated at slow frequency but in tandem. This suggests that the transformation of breast tissue from precancer to cancer involves a stepwise trajectory of gaining driver mutations which promote clonal sweeps. *PIK3CA* and *TP53* emerged as the most significantly mutated genes in breast cancer [26, 27]. Specifically, activating mutations in *PIK3CA* are found in approximately 40% of patients whilst *TP53* mutants are found in about 30% of all breast cancers.

At present, somatic driver mutations in normal breast tissue have not been described. There exists equipoise within the literature concerning the association between somatic driver mutations in normal tissue and future cancer risk. A 2018 study conducted to examine the association of somatic genetic variations to breast cancer onset revealed that somatic mutations detected in benign breast disease (BBD) tissue had no consequential effect on breast cancer risk [28]. Another longitudinal study revealed that somatic mutations were more frequent in BBD tissues in women who did not develop breast cancer within a 16-year observational window [29]. Intriguingly, the authors found that most mutated genes in BBD tissue of women who did not go on to develop breast cancer were related to cellular integrity and DNA repair (*MLH1, MSH2, PMS1, BRIP1* and *FAM175A)*. The authors postulated that these mutations could lead to site specific DNA damage responses that trigger the innate immune system and cellular clearance. The recruitment of immune cells promotes immune surveillance that protects against potential malignant transformation.

### Lung

The mutational burden in normal epithelium is strongly influenced not only by age, but also by smoking history. To this end, Yoshida et al. performed whole genome sequencing from single cells generated from the airway epithelium of the main or secondary bronchi [30]. Given the strong influence of smoking on somatic mutations, the authors grouped samples into never-smokers, ex-smokers and current smokers. Unsurprisingly, this resulted in a significant difference in the mutational burden between never smokers and current or ex-smokers. In never-smokers in whom an age-related accumulation of mutations is dominant, a mean of 22 single-based substitutions per cell per year was compared with a mean of 2330 in ex-smokers and 5300 in current smokers. The variability of mutations from cell to cell within the same individual also ranged from 290 per cell in never-smokers, compared with 2350 in ex-smokers and 2100 for current smokers. Intriguingly, individuals with any history of smoking exhibited a bimodal distribution of mutational burden, with one mode coinciding with never-smoker individuals, suggesting that in smokers, the additional mutational burden accrued from exposure to carcinogens was addictive to that of ongoing age-related mutations. It was therefore unsurprising that in such individuals, three unique mutational signatures, including SBS-4 and SBS-16, were found exclusively.

The authors identified *NOTCH1*, *TP53*, *ARID2*, *FAT1*, *PTEN*, *CHEK2*, and *ARID1A*, as being driver mutations found in normal bronchial epithelium. Driver mutations were found in 4–14% of cells from never-smokers, compared with 25% in current smokers. Overall, the authors computed a 2.1 fold increase in the frequency of driver mutations among individuals with any history of smoking compared with never-smokers. In terms of age, each decade of life brought about a 1.5 fold increase in the number of driver mutations per cell. In a separate study, TP53 mutations were detected in cell-free DNA from healthy controls, and it was deemed to pose serious challenge for early detection of small-cell lung cancer [31]. However, this potentially hinted on the possibility for pre-symptomatic detection for early intervention before disease onset.

### Liver

Whole genome sequencing of normal liver reflects the pathological transition of normal parenchyma to cirrhosis and finally cancer [32]. In normal liver parenchyma, sequencing revealed multiple clones which bore little genetic similarity to one another. Repeated insults to the liver result in the formation of cirrhotic nodules which are bound by fibrosis. As a result, nodules separated by fibrotic bands shared no mutations in spite of being adjacent to one another. Within each nodule, phylogeny suggested that most nodules were either monoclonal or oligoclonal, and possessed subclonal branches evident of ongoing mutational processes. The mutational signatures in cirrhotic liver reflect the diversity of exogenous insults to the liver. Signature 4 was observed in a number of samples, and is associated also with lung cancer from smokers. Another mutational signature observed was signature 24 which is associated to aflatoxin-B exposure, a known cause of hepatocellular cancer. In addition to these exogenous stimuli, clock-like signatures A and 5, as in many other tissue types, accounted for the bulk of mutational signatures and in combination, comprised 75% of the total mutational signature burden.

Driver mutations present in the liver include *ACVR2A*, *ARID2*, *ARID1A*, *TSC*, and *ALB* [33–35]. In general, driver mutations were rare, and comprised approximately 3% of all sequenced samples. Copy-number alterations appear to be a more significant occurrence in normal liver compared with other tissues, and included the presence of loss of chromosome 22 and 8p, and gain of chromosome 8q. It is therefore unsurprising that chromothripsis appears to be an ongoing process in chronic liver disease, as evidenced by 1–2% of clones bearing evidence of this multiple rearrangement event.

### Oesophagus

The oesophagus is frequently and directly exposed to extrinsic mutagens like alcohol or tobacco smoke. The mutational signatures present in the oesophagus reflect both age-related (SBS 1, 5) and exposure to tobacco smoke (SBS 4). Another mutational signature, SBS 16, of hitherto unknown aetiology has been described. To circumvent these normalizing mutagens, the epithelial surface is regularly sloughed and has a rapid cellular turnover [36]. Accelerated cell division makes it vulnerable to accruing somatic mutations at an alarming rate. Martincorena et al. performed ultradeep targeted gene sequencing of normal esophageal epithelium from non-oesophagus related deceased donors aged between 20 to 75 years old [37]. In their study, they found that the number of mutations was proportional to age. In the third decade of life, esophageal epithelial cells harbour an average of several hundred mutations per cell, increasing to more than 2000 by the seventh decade. Notably, they also discovered an over-representation of *NOTCH* family mutations in normal tissue. In particular, *NOTCH1* mutation was highly prevalent in normal aging oesophageal epithelium, and was found in 12–80% of cells. *TP53* is another cancer-associated mutation which was high prevalent in normal oesophageal cells (2–37%) [37]. These finding have since been mirrored in several other studies [19, 38, 39].

This high prevalence of *NOTCH1* mutations in normal tissue and absence in cancer represents a cryptic phenomenon. Multiple studies have described *NOTCH1* as a tumor suppressor gene [40, 41]. Recently, Abby et al. provided evidence that described the role of *NOTCH1* in healthy esophageal tissue [39]. In their study, they demonstrated that *NOTCH1* mutations in normal esophageal epithelium confer a beneficial effect due to accelerated clonal expansion, allowing *NOTCH1* mutant cells to colonize the epithelium while maintaining normal cellular function and behavior. Intriguingly, the authors also demonstrated that mutant *NOTCH1* is detrimental to cancer growth, which could explain their relative lack in oesophageal cancer. One possible explanation for this discordance is the occurrence of an immunogenic bottleneck in the early stage of oesophageal cancer development [42], allowing only cells with advantageous wild type *NOTCH1* to survive and expand. Regardless, these findings point towards our incomplete understanding of cancer evolution from normalcy to cancer.

### Small intestine

The small intestine is a unique organ because although it bears similarity to the crypt structure of the large intestine, the incidence of cancer is markedly reduced, and accounts for only 4% of gastrointestinal tract cancers [43]. On sequencing individual crypts, the most prevalent mutational signatures were SBS 1,5 and 18 [12]. SBS 1 and 5 are clock-like mutational signatures which we have described earlier. SBS 18 is characterized by C > A substitutions and is associated with the production of reactive oxygen species-induced DNA damage. A range of other mutational signatures were found in a subset of tissues, and were therefore considered to be sporadic in nature. These include SBS 17b and 35, which are associated with chemotherapeutic agents, SBS 41, which is of hitherto unknown etiology, and SBS 88 which is associated with colibactins produced by *Escherichia coli* in the gut microbiome [44]. SBS 2 and 13 were of particular interest as these accounted for a greater proportion of the mutational signature burden in small intestine compared with the large intestine. SBS2 and 13 contributed to 11% of the mutational burden, and were found in 22 out of 39 individuals. This mutational signature is associated with APOBEC mutagenesis. Driver mutations which were found in the small intestine include *FBXW7*, *ERBB2*, and *PIK3CA*. In addition, heterozygous truncating mutations were found in *RB1*, *FBXO11*, *FAT1*, *KMT2D*, *KMD6A*, *ACVR2A*, and *ZFHX3*.

### Colon

Similar to the small intestine, cells in the colon are organized into crypts, which form a clonal unit borne out of stem cell competition at the base of each crypt. In normal colonic epithelium, eleven signatures, comprising three single base substitutions, four double base substitutions and 3 indels, accounted for 85% of the mutational burden [45]. Single-base substitutions of note included SBS 1, 5 and 18, all of which are clock-like and exhibit a linear relationship with age. Intriguingly, the mutational burden for all three SBSs differed based on anatomic location along the colon. For SBS1, mean mutation rate across individuals ranged from 16.8 mutations per year (95% confidence interval 15.2–18.3) in the ascending colon and cecum, to 12.8 mutations per year (95% CI, 10.6–14.9) in the descending and sigmoid colon. Overall, the average mutation rate for SBS 1, 5 and 18 was 43.6 mutations per crypt per year.

Driver mutations present in the normal colon included truncating mutations in *STAG2* and *AXIN2*, and hotspot mutations in *PIK3CA* (E542K, R38H), *ERRB2* (R678Q, V842I, T862A), *ERBB3* (R475W, R667L), and *FBXW7* (R505C, R658Q) [45]. Taken together, these mutations were present in about 1% of normal colorectal crypts. A surprising observation from this list of mutations in normal tissue is the absence of known driver mutations in CRC such as *APC*, or *KRAS*, raising questions concerning the role of normal driver mutations in oncogenesis, if any, and whether such normal somatic mutations impact upon the later acquisition of CRC driver mutations.

The landscape of somatic mutagenesis in normal colonic tissue in patients with germline mutations have also been undertaken. Patients with germline *MUTYH* mutations have an elevated adenoma formation rate, and consequently are at increased risk of CRC in a clinical syndrome known as *MUTYH*-associated polyposis (MAP). MUTYH is a protein associated with the base excision repair (BER) pathway, such that defects in MUTYH result in elevated C > A transversions [46]. In patients with germline *MUTYH* mutations, SBS 1, 5, 18, and 36 mutational signatures were identified. The burden of SBS 1 and 5 was acquired at a similar rate as in wildtype individuals, such that the increased mutational burden observed in patients with germline *MUTYH* mutations could be accounted for by SBS 18 and 36 [47].

Individuals with germline mutations in *POLE* and *POLD1* develop the clinical syndrome known as polymerase proofreading-associated polyposis (PPAP) and are also characterized by early onset CRC and endometrial cancer. Pol ε and Pol δ are responsible for identifying and removing mismatched base pairs during DNA replication and dysfunction results in a high burden of SBS mutational signatures. As in individuals with germline *MUTYH* mutations, SBS 1, 5 were again detected at a similar burden as wildtype individuals. The effect of germline mutations in *POLE* and *POLD1* was manifested in SBS 10 and 28 [48].

Chronic inflammation of the colon, as in the setting of inflammatory bowel disease (IBD), is another known etiology for CRC. To evaluate the mutational signature burden in patients with inflammatory bowel disease, Olafsson et al. performed whole-genome sequencing of crypts originating from patients with inflammatory bowel disease, and identified similar mutational signatures to normal non-inflammed colon [25]. SBS 1, 5 and 18 again accounted for more than 80% of the mutational signature burden. Some of the remaining 20% could be accounted for by treatment effects such as exposure to purine-treatment in the form of azathioprine (SBS 32). Importantly, although there was broad concordance between the mutational signature, the somatic mutations present in inflamed but non-cancerous colonic tissue differed from those found in normal colon. In IBD colon, somatic driver mutations included *ARID1A*, *FBXW7*, *PIGR*, and *ZC3H12A*. *AXIN2* and *STAG2* found in normal colon was not found in IBD colon, while *PIGR* and *ZC3H12A* were not found in normal colon. In a rare example of a mechanistic validation of the role of these driver mutations in disease, Nanki et al. demonstrated the critical role of IL17 signaling in relation to *PIGR* and *ZC3H12A* [49]. The authors established that mutations in the IL17 signaling pathway abrogated Il17-mediated apoptosis, allowing cells carrying the mutation to expand clonally in spite of ongoing inflammation.

### Kidney

Using whole genome sequencing, Franco et al. successfully aligned 8 signatures by comparing 192 tissue-matched tumor samples to 161 healthy kidney samples, of which 4 signatures (SBS1, 3, 5, 8) were found ubiquitously in both healthy and malignant samples and were linearly influenced by age [50]. This finding suggested that these mutational signatures were fundamental for malignant transformation. Notably, their analysis delineated kidney tissues into specific cell types, through which they uncovered mutagen-specific signatures which allowed us to gain environment-related insights into the mechanisms of mutagenesis in renal cells. For example, kidney epidermal cells harbour a high prevalence of the SBS7a signature which is associated with UV light exposure. WGS has also enabled a precise mapping of the transformation trajectory of renal cells. Most notably, Young et al. identified a specific population of epithelial cells from proximal convoluted tubular cell as potential precursors of clear cell renal cell carcinoma and papillary renal cell carcinoma and characterized its unique transcriptional features termed “PT1 signature” marked by *VCAM1*, and *SLC17A3* expression [51].

### Bladder

Prior to the mutational screen of the bladder urothelium, it has been speculated that the acquisition and accumulation of somatic mutations correlate strongly to rapid cellular divisions. This observation arose from mutational screens of skin or esophagus where both organs have rapid doubling times and a significant population of cells accumulating mutations (30% and 50% respectively). Instead, bladder urothelium demonstrated the ability to accrue high mutational burden, with approximately 19% of bladder epithelial cells harbouring driver mutations [52], despite being one of the slowest cycling epithelial cell types in the body with a turnover rate of around 200 days [53]. Due to its constant exposure to carcinogens and mutagens in urine, bladder cancers have one the highest mutation burdens among major cancer types.

Unlike the oesophagus or colon, positively selected mutations in normal bladder were also found in bladder cancer. In total, 17 genes identified in a screen were driver mutations in bladder cancer [54]. These 17 genes can be classified into three distinct clusters based on their functions, namely the RTK-Ras-PI3K pathway, the p53-Rb pathway and chromatic remodeling pathway. Of note, four of the top six most-mutated driver genes (*KMT2D, KDM6A, ARID1A* and *EP300*) in the normal bladder are chromatin remodeling genes. This suggests that mutations in chromatin remodeling genes, though pervasive and selectively advantageous, are insufficient to initiate cancer transformation on its own. Three groups of mutational signatures dominated the mutational landscape in this study. These included age-related changes (SBS 1 and 5), APOBEC3 cytidine deaminase mediated mutagenesis (SBS 2, and 13), and mutagenesis by the mutagen aristocholic acid (SBS 22).

### Prostate

Parr et al. described the presence of mitochondrial DNA mutations in three regions of patients with prostate cancer—malignant tumour, adjacent benign and distant benign tissue, performing whole mitochondrial gene sequencing on these three tissue sites [55]. The authors focused on the 13 genes involved in oxidative phosphorylation as well as hypervariable segments 1 and 2 (HV 1 and 2). The authors noted that mitochondrial gene mutations were present in all three sites, including distant benign, in 66.7% (16 of 24) samples. In a further comparison between tissue from malignant samples and age-matched benign prostate tissue, the authors noted increased mutational burden in the coding regions of malignant samples, but no statistically significant difference in the non-coding regions, suggesting that benign prostatic tissue begin by acquiring mutations in non-coding regions before a malignant switch is observed in tandem with the acquisition of mutations in coding regions.

The primacy of mutation accumulation in non-coding regions could explain why somatic driver mutations are rare in adult prostatic epithelium. Focusing on normal prostatic epithelium, only one driver mutation in *FOXA1* was observed [18], even though there was a persistent clock-like accumulation of mutations at an average rate of 16.4 per year per mutant clone, large contributed by the mutational signatures SBS 1, 5, and 18. These findings have profound implications on stem cell dynamics in the developing and adult prostatic epithelium, and suggests that normal prostatic epithelium maintains a tight architecture which limits migration of clones. In fact, each clonal unit is populated by its own stem and progenitor cells. Whether cancer arises as a result of breakages in these tight-linked dependencies remains to be explored.

### Endometrium

Initial interest in the presence of driver mutations in benign endometriotic tissue stemmed from investigations into endometriosis, a condition characterized by ectopic endometrial tissue which acquires tumour-like characteristics such as infiltration and growth along intraabdominal surfaces. In one study, cancer driver genes *ARID1A*, *PIK3CA*, *KRAS*, and *PPP2R1A* were identified in 21% of endometriotic lesions [56]. Another study confirmed the presence of the above cancer driver genes, and also found additional mutated genes such as *TAF1*, *SPEG*, *ACRC*, and *FAT1* [57]. Notably, *KRAS* and *PIK3CA* appear to be important in the initial stages of the evolutionary trajectory of endometriotic cells. Suda et al. demonstrated that *KRAS* and *PIK3CA* lesions carrying the same mutations could be found at disparate regions of an endometriotic lesion, and all with high variant allele frequencies (VAF), suggesting that all lesions share a common somatic progenitor cell. In addition, single-gland sequencing showed that *PIK3CA* mutations were present in more than one-third of all glands sequenced.

Given the prevailing hypothesis that endometriotic tissue derives from retrograde menstruation and deposition of endometriotic fragments into the peritoneal cavity, it was unsurprising that sequencing of normal uterine tissue revealed a similar spectrum of somatic mutations. Mutational signatures associated with somatic mutations comprised SBS 1, 5, 18, 23 and 40, as well as ID 1 [58]. Interestingly, this resulted in a high burden of somatic mutations present in a majority of normal endometrial glands [58, 59], with some glands even possessing more than four driver mutations. Phylogenetic analysis of these mutations highlighted that *KRAS*, *PIK3CA*, and *ZFHX3* mutations appeared to be acquired early in life, possibly as early as the first decade. This finding could explain a trend showing a higher frequency of *KRAS* mutations in normal endometrial gland compared to endometrial cancer (28% vs 19%; *p* = 0.0728) [60]. As in endometriotic tissue, *PIK3CA* was observed to be the most frequently mutated cancer driver gene in normal endometrial tissue. Furthermore, both *PIK3CA* and *KRAS*, together with other somatic mutations found in normal endometrial tissue were found to be under strong positive selection pressure based on dN/dS ratios. Intriguingly, Yamaguchi et al. used a tissue clearing technique in combination with light-sheet fluorescence microscopy to uncover the horizontal expansion of endometrial glands along the muscular layer of the uterus, giving rise to glands at separate regions of the uterus with related patterns of somatic mutations [59].

### Summary

**Fig. 1:**
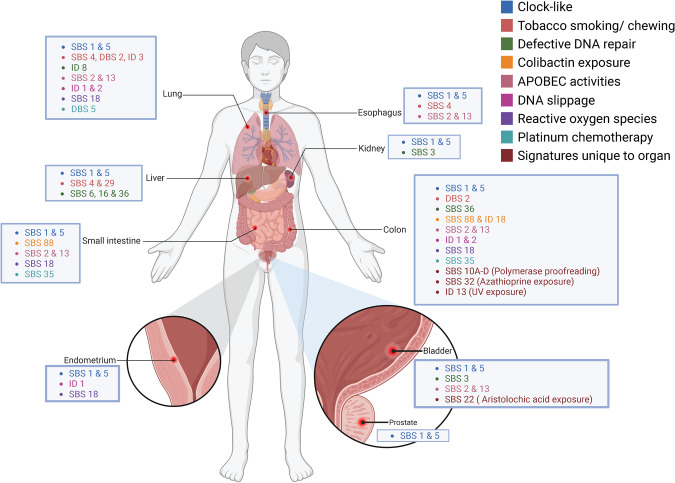
Summary of mutational signatures and associated aetiology for different solid and hollow viscus organs. Clock-like mutational signatures (SBS 1 and 5) are ubiquitous in all organs. Other mutational signatures, such as those related to colibactin exposure (SBS 88) appear limited to the small intestine and colon. This figure demonstrates the landscape of somatic driver mutations found in normal tissues and can be used to visualize commonalities and differences among various tissue types.

Here, we provide an in-depth review concerning the mutational signatures and somatic driver mutations present in a range of solid and hollow viscus organs. Table 1 summarises the mutational signature and the driver mutations present in each organ as described above, while Fig. 1 compiles mutational signatures and their associated aetiologies. Whenever possible, we included mutations which were noted to be more prevalent in normal tissues than in the corresponding cancer for that tissue. This highlights genes which appear to undergo an unusual dynamic, in that driver mutations which had acquired a fitness advantage in normal tissues must have undergone a change in its relative fitness, resulting in its diminution in cancer. We also highlight driver mutations which appear unique to a specific organ, as this could highlight tissue-specific circumstances. Admittedly, analysing the landscape of somatic driver mutations in this way throws up more questions than answers. For example, while it may be argued that the clock-like signatures SBS 1, and 5, are generally ubiquitous across all tissue types, there is no driver mutation in normal tissue which is common across all tissue types, implying that one cannot draw direct conclusions about the role of mutational signatures per se without simultaneously considering the somatic driver mutations which have been impacted.

**Table 1:** Summary of the pattern of mutational signatures and somatic mutations for different types of normal tissue.

Organ	Tissue	Mutational signatures	Somatic mutations under positive selection	Mutations more frequent in normal than cancer	Mutations unique to this organ	References
Lung	Normal bronchial epithelium from smokers, and ex-smokers	SBS 1, 2, 4, 5, 13, 16, 18, A, B DBS 2, 4, 5, 6, 11, C ID 1, 2, 3, 5, 8	*ARID1A, ARID2, CHEK2, FAT1, NOTCH1, PTEN, TP53*	-	-	[30]
Liver	Normal hepatocytes	SBS 5, A	*ACVR2A, ALB*	-	*ALB*	[32]
	Cirrhotic liver parenchyma	SBS 1, 5, 12, 16, 40, A, D	*ACVR2A, ALB*	-	*ALB*	[32]
	Normal hepatocytes	SBS 5, 18, 36	*-*	-	-	[33]
	Non-dysplastic hepatocytes from patients with CLD	SBS 4, 6, 15, 29	*ALB, ALMS1, APOB, APOBR, ARID1A, ARID2, AUTS2, CDH8, CHD2, CLASP1, COL22A1, DSPP, EP400, FBN2, IGFN1, KMT2D, LOR, MKI67, NF1, PAPPA2, PKD1, PKHD1, PPARGC1B, STARD9, SUZ12, TP53*	*ALMS1, KMT2D, PKHD1*	*ALB, ALMS1, APOB, APOBR, AUTS2, CDH8, CHD2, CLASP1, COL22A1, DSPP, EP400, FBN2, IGFN1, LOR, MKI67, NF1, PAPPA2, PKD1, PKHD1, PPARGC1B, STARD9, SUZ12*	[34]
	Cirrhotic liver parenchyma	T > A in a CTG context	*ACVR2A, ALB, ATP6V0C, CIDEB, FOXO1, GPAM, NEAT1, RN7SK, TNRC6B*	*FOXO1, GPAM, TNRC6B*	*ALB, ATP6V0C, CIDEB, FOXO1, GPAM, NEAT1, RN7SK, TNRC6B*	[35]
Oesophagus	Normal oesophageal squamous epithelium	SBS 1, 5, 16	*AJUBA, ARID1A, ARID2, CCND1, CUL3, FAT1, KMT2D, NFE2L2, NOTCH1, NOTCH2, NOTCH3, PIK3CA, TP53, TP63*	*NOTCH1*	*AJUBA, CCND1, CUL3, NFE2L2, NOTCH3, TP63*	[37]
	Normal oesophageal squamous epithelium	SBS 1, 2, 4, 13, 16	*CHEK2, FAT1, NOTCH1, NOTCH2, NOTCH3, PAX9, PIK3CA, PPM1D, TP53, ZFP36L2*	*CHEK2, FAT1, NOTCH1, NOTCH2, NOTCH3, PPM1D, ZFP36L2*	*NOTCH3, PAX9, PPM1D, ZFP36L2*	[19]
Small intestine	Normal small intestinal epithelium	SBS 1, 2, 5, 13, 17b, 18, 35, 40, 41, 88	*ACVR2A, ERBB2, FAT1, FBXO11, FBXW7, KMD6A, KMT2D, PIK3CA, RB1, ZFHX3*	-	*FBXO11, KMD6A*	[12]
Colon	Normal colonic epithelium	SBS 1, 2, 5, 13, 18, 88, 89, C, D DBS 2, 4, 6, 8, 9, 11 ID 1, 2, 5, 18, B	*AXIN2, ERBB2, ERBB3, FBXW7, PIK3CA, STAG2*	*AXIN2, ERBB2, ERBB3, FBXW7, PIK3CA, STAG2*	*AXIN2*	[45]
	Organoids derived from ulcerative colitis-infammed epithelia	-	*ARID1A, IL17RA, NFKBIZ, PIGR, ZC3H12A*	*ARID1A, IL17RA, NFKBIZ, PIGR, ZC3H12A*	*IL17RA, NFKBIZ, PIGR, ZC3H12A*	[49]
	Ulcerative and crohn’s disease affected colonic epithelium	SBS 1, 2, 5, 13, 17a, 17b, 18, 32, 35, 88, 89, C ID 1, 2, 14, 18, B	*ARID1A,* *FBXW7,* *PIGR,* *ZC3H12A*	*ARID1A,* *FBXW7,* *PIGR,* *ZC3H12A*	*PIGR, ZC3H12A*	[25]
	Colorectal, ileal and duodenal epithelia from individuals with exonuclease domain mutations in *POLE* or *POLD1*	SBS 1, 5, 10a, 10b, 10c, 10d, 17a, 17b, 28, 35, 88, 89 ID 1	*AMER1, APC, ARID1A, ATRX, BCOR, CDK12, FBXW7, KMT2C, PIK3R1, UBR5*	*-*	*AMER1, APC, ATRX, BCOR, CDK12, UBR5*	[48]
	MAP-affected colonic epithelia	SBS 1, 5, 18, 36, 88	*-*	*-*	-	[47]
Kidney	Renal proximal tubule cells	SBS 1, 3, 5, 8	*-*	-	-	[50]
Bladder	Urothelium	SBS 2, 3, A, B, C	*ARID1A, CDKN1A, CREBBP, ELF3, EP300, ERCC2, FOXQ1, GNA13, KDM6A, KLF5, KMT2D, NOTCH2, PTEN, RBM10, RHOA, STAG2, ZFP36L1*	-	*CDKN1A, CREBBP, ELF3, EP300, ERCC2, FOXQ1, GNA13, KDM6A, KLF5, RBM10, RHOA, ZFP36L1*	[52]
	Urothelium from bladder and ureter	SBS 1, 2, 5, 13, 22	*ARID1A, CDKN1A, CHEK2, CREBBP, ELF3, EP300, ERCC2, FGFR3, FOXQ1, KDM6A, KMT2D, PIK3CA, RB1, RHOB, STAG2, TP53, TSC1, UTY, ZFP36L1*	-	*CDKN1A, CREBBP, ELF3, EP300, ERCC2, FGFR3, FOXQ1, KDM6A, RHOB, TSC1, UTY, ZFP36L1*	[54]
Prostate	Normal prostatic epithelium	SBS 1, 5, 40	*FOXA1*	-	*FOXA1*	[18]
Endometrium	Endometriotic tissue		*ARID1A, KRAS, PIK3CA, PPP2R1A*		*PPP2R1A*	[56]
	Endometriotic tissue		*ACRC, ARID1A, FAT1, KRAS, PIK3CA, SPEG, TAF1*		*ACRC, SPEG, TAF1*	[57]
	Normal endometrium		*AKT1, ERBB2, FGFR2, KRAS, NRAS, PIK3CA, PTEN*	*KRAS*	*AKT1, NRAS*	[60]
	Normal endometrium	SBS 1, 5, 18, 23, 40 ID 1	*ARHGAP35, CHD4, ERBB2, ERBB3, FBXW7, FOXA2, KRAS, PIK3CA, PIK3R1, PPP2R1A, SPOP, ZFHX3*		*ARHGAP35, CHD4, FOXA2, PPP2R1A, SPOP*	[58]
	Normal endometrium	SBS 1, 5, 18	*ARHGAP35, ARID1A, ARID5B, FBXW7, FGFR2, KMT2C, KRAS, PIK3CA, PIK3R1, PLXNB2, PPP2R1A, PTEN, TAF1, TP53, ZFHX3*		*ARHGAP35, ARID5B, PLXNB2, PPP2R1A, TAF1*	[59]

Perhaps given the lack of unanimity across tissues, it is unsurprising that mutational events, and the proportion of normal cells in each organ which harbour a somatic driver mutation varies widely (Table 2). In particular, there appears to be no relationship between the mutation rate in tissues and the proportion of normal cells with somatic driver mutations. The endometrium has one of the lower mutational rates at 29 mutations per gland, yet, close to 60% of cells have a somatic driver mutation. In contrast, smokers possess a high mutational burden at 5300 mutations per cell, yet only 25% of cells harbour a somatic driver mutation. Furthermore, there is a disconnect between the frequency of somatic driver mutations in normal tissue, and the incidence rates for cancer. For example, although approximately 5% of cells in the normal colon possess somatic driver mutations, compared to 90% in the oesophagus, yet the 2019 global age-standardised incidence rates for oesophageal cancer was 6.51 per 100,000 compared with 26.71 for colorectal cancer [61]. The disparity in the frequency of normal cells with driver mutations and the cancer incidence rates demonstrates our deficiency in understanding the role of driver mutations in normal tissues.

**Table 2:** Summary of the mutational rates and proportion of tissue which possess somatic mutations in different types of normal tissue.

Organ	Tissue	Proportion with somatic mutations	Mean number of mutations per basepair per year	Reference
Lung	Normal bronchial epithelium	Never-smokers: 4–14% Current smokers: 25%	Never-smokers: 22 per cell Ex-smokers: 2330 per cell Smokers: 5300 per cell	[30]
Liver	Cirrhotic liver parenchyma	-	33 per diploid genome	[32]
	Normal hepatocytes	-	Liver stem cells: 11 per cell per mitosis Hepatocytes: 21 per cell per mitosis	[33]
Oesophagus	Normal oesophageal squamous epithelium	24/25, 96%	41.5 per genome	[19]
Small intestine	Normal small intestinal epithelium	-	Duodenum: 51 per crypt Jejunum: 50 per crypt Ileum: 42 per crypt	[12]
Colon	Normal colonic epithelium	26/445, 5.8%	43.6 per crypt	[45]
	Ulcerative and crohn’s disease affected colonic epithelium	-	95 per crypt	[25]
	Colorectal, ileal and duodenal epithelium from individuals with exonuclease domain mutations in POLE or POLD1	20/109, 18.3%	*POLE* L424V: 331 per crypt *POLD1* S478N: 152 *POLD1* D316N and L474P: 58	[48]
	MAP-affected colonic epithelium	22 / 144, 15%	MUTYH Y179C: 177 MUTYH Y104*: 193 MUTYH G286E: 145	[47]
Kidney	Renal proximal tubule cells	-	11.7–55.6 per genome	[50]
Bladder	Normal urothelium	-	1879 per genome	[52]
	Normal urothelium from bladder and kidney	-	2.2 per megabase DNA	[54]
Prostate	Benign prostatic epithelium in patients with prostatic cancer (mitochondrial DNA)	Adjacent benign: 19/24, 79.2% Distant benign: 22/24, 91.7%	-	[55]
	Normal prostate epithelium	-	16.4 per clone	[18]
Endometrium	Normal endometrium	1 driver: 147/257 glands, 57.2% 2 drivers: 42/257, 16.3% ≥4 drivers: 5/257, 1.9%	29 per gland	[58]
	Normal endometrium	551/891 glands		[59]

## Implications and future directions

**Fig. 2:**
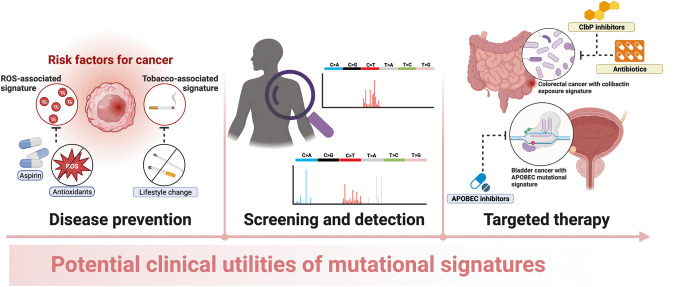
Schematic describing potential clinical utilities arising from mutational signatures. Further research into mutational signatures could translate into therapeutic potential. One area is disease prevention. Understanding aetiologic associations between mutational signature and cancer can lead to lifestyle modifications which can reduce the trigger for mutations. Screening and detection are other areas in which mutational signatures may be harnessed. Here, cell-free approaches may be combined with the detection of mutational signatures arising from normal tissue in peripheral blood, bringing forward the detection of future malignant risk by decades from what is presently achievable. Finally, targetted therapy could be used to specifically reverse or slow down mutagenesis if the burden of mutational signatures in an individual is known. Together, these present potential therapies which could reduce overall incidence of cancer.

Here, we describe three areas in which the above-described landscape of somatic mutations can be taken advantage of in therapeutic settings (Fig. 2).

### Screening

Recent evidence from single-cell sequencing of healthy colon, polyps and colorectal cancer suggests that a continuum of epigenetic and transcriptional changes occur which can be used to trace the gradual loss of homeostasis which heralds cancer [62]. Further evidence from multi-regional sequencing of cancer revealed the same subclonal mutations present at geographically discontinuous parts of a tumour, suggesting that some cells lost normal cell adhesion and gained increased mobility early in the genesis of the tumour [63]. Together these features suggest that somatic mutations acquired while tissues remain phenotypically normal could be harbingers of future cancer.

This raises questions surrounding present screening practices. Screening programs have been developed for a range of cancers including breast, colorectum and the cervix. In all examples, screening requires the detection of an abnormal lesion—an opacity on mammogram for breast cancer, a polyp in colorectal cancer, or abnormal squamous epithelia in the cervix. In these examples, the detection of a lesion at screening suggests that cancer drivers have already reached clonal proportions within the tissue. In the colon, more than 80% of polyps possess clonal *APC* loss [64]. Given that individuals spend a far greater period of time in normalcy than in the precancerous or cancerous phases, and combined with the possibility that cells acquire mutations during normalcy which could have implications for future cancer, screening should be focused not on the detection of premalignant lesions, but instead on the detection of individuals who already possess potentially harmful somatic mutations in normal tissue. An even greater benefit would be seen in the many cancers in which no effective screening modality exist, such as in the oesophagus, bladder or liver.

### Novel application of cell free DNA mutation profiling

A specific modality of screening could harness current technology of high sensitivity mutational profiling using cell free DNA. Cell free DNA is attractive in cancer treatment because it is minimally invasive, and yet possesses the requisite sensitivity to detect somatic tumour mutations [65]. This technology could potentially be even more attractive for cancer screening because patients are often reluctant to undergo invasive investigations when they perceive themselves to be healthy. A blood test could be used to identify somatic mutations or mutational signatures. One challenging aspect would be consequently identifying the organ of source for the somatic mutations. In such a case, the pattern of mutational signature and somatic mutations could be aligned to existing known mutational patterns in normal tissue, and used to narrow down organ systems which can then be screened with a targeted approach. Clinical trials will need to be performed to fully implement such a modality of screening into clinical practice.

### The potential for targetted therapy

Tissues with a high mutational burden which have yet to exhibit malignant phenotypes could be an indication for targetted therapy. Since mutational signatures ultimately manifest in the accrual of somatic mutations, one avenue where therapy could be applied would be with the aim of slowing down the accumulation of mutations. Here, it is helpful to consider the associations between aetiology and mutational signatures, and to formulate therapy to target specific effects of each mutational signature.

In the small intestine and colon, colibactin is associated with mutational signature SBS 88. Colibactin is produced by *Escherichia coli*, and is a small molecule which alkylates DNA, resulting in DNA adducts and double-stranded DNA breaks [66]. Therapeutics targeted at either inhibiting the production of colibactin, or neutralizing its alkylating effect could potentially mitigate against the effects of colibactin. Recently, bacteria targeted by colibactin express a colibactin resistance protein which has been shown to protect certain forms of bacteria from the deleterious effects of colibactin [67]. This could potentially represent a treatment modality administered endoscopically to patients detected with a high burden of mutation.

Similarly, APOBEC cytidine deaminases have been implicated in SBS 2 and 13, and are associated in a wide range of normal tissue including the lung, oesophagus, small intestine, colon and bladder. There has been renewed interest in understanding the effects of the APOBEC cytidine deaminases in humans. Previously, the APOBEC3 family was thought to be critical in innate immune restriction of retroviruses and retroelements [68]. In mice, however, APOBEC3 inhibition demonstrated that increased susceptibility to retroviruses did not however affect cellular viability, and there was likely redundancy among pathways which could compensate for the decrease in APOBEC activity [69]. These early steps could pave the way for therapeutic intervention using APOBEC inhibitors in patients with a heavy burden of the mutational signature.

## Conclusion

Multiple studies have demonstrated the spectrum of mutational signatures and driver mutations in a range of tissues. Here, we have critically analysed the literature and attempted to draw commonalities and highlight differences between tissue types, attempting to make sense of the complex landscape of driver mutations in normal tissue, and how this could relate to future cancer. The chaotic landscape of somatic driver mutations suggests that a unified understanding of the role of somatic mutations in normal tissue is currently beyond reach. Instead, sufficient tissue-specific characteristics abound that warrant approaching this matter from a tissue-specific manner. The role of driver mutations as expressed in tumour suppressor genes and protooncogenes is foundational to our understanding of cancer. Yet, in normal tissues, few studies have attempted to mechanistically unpick the role of individual mutations in normal tissue, and must be the focus of current research. The potential implications to how we screen and treat cancer are enormous.
